# Supplementary material for: Local Landscapes, Evolving Minds: Mechanisms of Neighbourhood Influence on Dual-State Mental Health Trajectories in Adolescence
Source: Int J Environ Res Public Health. 2025 Jun 17;22(6):951. doi: 10.3390/ijerph22060951 (PMC12199192; doi:10.3390/ijerph22060951)

Local Landscapes, Evolving Minds: Mechanisms of Neighbourhood Influence on Dual-State Mental Health Trajectories in Adolescence

(Supplementary Material)

Table of Contents

Representativeness of the Sample

Table S1. Demographic characteristics of the analytical sample against Greater Manchester (GM) and national population statistics 5

Extended Descriptive Statistics

Table S2. Extended Descriptive Statistics 7

Adjusted Analysis (Main Analysis Adjusted for Gender and Ethnicity)

Table S3. Associations Between Aspects of Community Wellbeing and Mental Health Trajectories, Adjusting for Gender and Ethnicity 11

Table S4. Total, Direct and Indirect Effects of Neighbourhood Deprivation and Community Wellbeing on Life Satisfaction Trajectories, Adjusting for Gender and Ethnicity........ 12

Table S5. Total, Direct and Indirect Effects of Neighbourhood Deprivation and Community Wellbeing on Emotional Difficulties Trajectories, Adjusting for Gender and Ethnicity 13

Model Fit Criteria

Table S6. Model Fit Statistics for Enumeration of Life Satisfaction Trajectories (N = 27,009)15

Table S7. Model Fit Statistics for Enumeration of Emotional Difficulties Trajectories (N = 26,461) 16

a and b Pathways for Main Analysis

Table S8. Standardised Regression Coefficients Between Neighbourhood Deprivation and Aspects of Community Wellbeing with 95% Confidence Intervals 18

Table S9. Associations Between Aspects of Community Wellbeing and Mental Health Trajectories 19

Mapping the Co-op Wellbeing Index Across Greater Manchester Seamless Locales

Figure S1. Heat map of Greater Manchester: Relationships and Trust 21

Figure S2. Heat map of Greater Manchester: Equality 21

Figure S3. Heat map of Greater Manchester: Voice and Participation 22

Figure S4. Heat map of Greater Manchester: Economy, Work and Employment 22

Figure S5. Heat map of Greater Manchester: Health 23

Figure S6. Heat map of Greater Manchester: Education and Learning 23

Figure S7. Heat map of Greater Manchester: Culture, Heritage and Leisure 24

Figure S8. Heat map of Greater Manchester: Housing, Space and Environment 24

Figure S9. Heat map of Greater Manchester: Transport, Mobility and Connectivity 25

Alternative Model Solutions (Probability Plots)

Figure S10. Life Satisfaction - Linear Growth with Variance Held Equal Across Classes 27

Figure S11. Life Satisfaction – Non-Linear Growth with Variance Held Equal Across Classes28

Figure S12. Life Satisfaction – Linear Growth with Variance of Slope Factor Fixed at Zero .. 29

Figure S13. Life Satisfaction – Non-Linear Growth with Variance of Slope Factor Fixed at Zero 30

Figure S14. Emotional Difficulties - Linear Growth with Variance Held Equal Across Classes31

Figure S15. Emotional Difficulties – Non-Linear Growth with Variance Held Equal Across Classes32

Figure S16. Emotional Difficulties – Linear Growth with Variance of Slope Factor Fixed at Zero 33

Figure S17. Emotional Difficulties – Non-Linear Growth with Variance of Slope Factor Fixed at Zero

.... 34

Representativeness of the Sample

Table S1. Demographic characteristics of the analytical sample against Greater Manchester (GM) and national population statistics

EAL English as an additional language; FSM Free School Meal Eligibility over the last six years; SEN Special

	Sex (%)	FSM (%)	EAL (%)	SEN (%)	Ethnicity (%)
Analytical Sample	F = 50.1<br>M = 49.9	No = 72.2<br>Yes = 27.8	No = 79.1<br>Yes = 20.9	No = 83.6<br>Yes = 16.4	AOEG = 2.5<br>Asian = 17.0<br>Black = 6.4<br>Chinese = 1.0<br>Mixed = 6.4<br>White = 66.6
GM Population of Year 8 Pupils [a]	F = 48.7<br>M = 51.3	No = 64.4<br>Yes = 35.6	*No = 76.9<br>*Yes = 22.7	No = 81.1<br>Yes = 18.9	*AOEG = 3.0<br>*Asian = 17.6<br>*Black = 7.1<br>*Chinese = 1.1<br>*Mixed = 6.5<br>*White = 63.1
National Statistics for Year 8 Pupils [a]	F = 48.7<br>M = 51.3	No = 71.0<br>Yes = 29.0	*No = 81.2<br>*Yes = 18.1	No = 81.5<br>Yes = 18.5	*AOEG = 2.3<br>*Asian = 12.2<br>*Black = 6.3<br>*Chinese = 0.7<br>*Mixed = 6.5<br>*White = 70.0

Educational Needs

[a] *National statistics data gathered in the academic year 2022/23: here*

* Proportions for Year 8 were unavailable, so statistics represent proportions across years in state-funded secondary schools

Extended Descriptive Statistics

Table S2. Extended Descriptive Statistics

Variable		Life Satisfaction Overall	Life Satisfaction Improving	Life Satisfaction Deteriorating	Life Satisfaction Low	Life Satisfaction High	Emotional Difficulties Overall	Emotional Difficulties Sub-Clinical	Emotional Difficulties Worsening	Emotional Difficulties Low
Sample Size *N*		27,009	2,358	1,704	3,770	19,177	26,461	10,137 (38.3)	2,113	14,211
(%)		(100)	(8.7)	(6.3)	(14.0)	(71.0)	(100)		(8.0)	(53.7)
Girls *(%)*		50.1	51.7	49.7	50.5	40.0	50.2	50.4	50.4	38.8
SEN (%)		16.7	15.4	14.6	17.7	11.4	16.1	13.7	16.2	11.3
FSM (%)		27.8	23.4	14.3	33.8	7.1	27.8	20.7	28.1	8.6
EAL (%)		20.9	10.1	7.1	26.6	10.9	20.9	9.5	20.9	10.6
Ethnicity	White *(%)*	66.5	81.4	83.5	59.2	58.8	66.7	81.2	66.5	59.9
	Any Other Ethnic Group *(%)*	2.6	1.7	1.3	3.1	0.4	2.5	1.7	2.5	1.1
	Asian *(%)*	17.1	9.5	7.5	21.3	1.5	17.0	8.9	17.2	1.6
	Black *(%)*	6.4	2.8	1.5	8.6	1.5	6.4	2.9	6.4	1.6
	Chinese *(%)*	1.0	0.3	1.2	0.8	31.2	1.0	0.7	0.9	29.7
	Mixed *(%)*	6.5	4.3	5.1	7.0	6.7	6.4	4.4	6.4	6.0
IMD %		66.8	56.7	37.6	79.2	37.0	66.8	54.4	67.3	35.6
Life Satisfaction / Emotional Diffs. 2021 (*Mean)*		6.9	3.0	7.9	3.8	7.9	6.6	8.7	10.9	4.5
Life Satisfaction / Emotional Diffs. 2022 (*Mean)*		6.6	5.8	4.3	3.9	7.6	6.6	8.8	12.1	4.1
Life Satisfaction / Emotional Diffs. 2023 (*Mean)*		6.6	7.5	2.2	4.0	7.4	6.3	9.2	16.7	2.5
Community Wellbeing Index										
Domain	Indicator (*Mean)*									
Relationships and Trust		.695	.662	.680	.702	.679	.695	.664	.696	.679
	Social Spaces	.912	.720	.901	.921	.688	.912	.721	.915	.691
	Prescence of Young Children	.766	.617	.655	.816	.536	.766	.617	.770	.513
	One Person Households, aged 50+	.352	.497	.353	.348	.573	.352	.505	.349	.565
	Proximity of Work to Home	.893	.872	.858	.907	.826	.892	.865	.893	.828
	Household Churn	.413	.367	.294	.463	.429	.412	.350	.414	.451
	Long-Term Health Status	.331	.378	.471	.272	.573	.332	.396	.330	.571

Crime in Locale per 10,000	.996	.994	.994	.997	.983	.996	.992	.996	.969
Crime in the Town Centre	.988	.986	.983	.990	.997	.988	.985	.988	.996
Equality	.544	.553	.502	.561	.462	.544	.542	.544	.480
Gap in House Prices	.714	.726	.522	.793	.577	.713	.699	.715	.597
Second Home Ownership	.719	.724	.796	.687	.693	.719	.710	.719	.732
Proximity to Independent Schools	.039	.095	.058	.030	.176	.039	.095	.038	.175
Gap in Qualifications	.546	.522	.536	.552	.359	.547	.498	.548	.396
Ethnic Minority Representation in Professional Occupations	.439	.458	.448	.435	.375	.440	.482	.439	.361
Relative Affluence	.707	.561	.378	.846	.251	.705	.531	.710	.266
Long-Term Housing Security	.473	.617	.641	.401	.705	.475	.621	.471	.725
Voice and Participation	.546	.524	.582	.532	.514	.547	.530	.547	.513
Voter Turnout	.098	.091	.195	.059	.117	.099	.103	.098	.115
Co-op Member Engagement	.543	.534	.563	.536	.420	.544	.538	.544	.413
Signing Petitions	.924	.877	.911	.930	.921	.924	.879	.925	.924
Economy, Work and Employment	.658	.661	.763	.615	.645	.659	.667	.658	.652
Proximity of Work to Home	.893	.872	.858	.907	.826	.892	.865	.893	.828
Hours Worked	.938	.851	.863	.971	.691	.937	.830	.940	.724
Household Income	.259	.382	.577	.125	.627	.260	.416	.256	.611
Vacant Commercial Units	.746	.989	.800	.718	.888	.746	.994	.742	.836
Free School Meals	.209	.253	.496	.091	.293	.210	.265	.208	.286
Unemployment	.123	.252	.306	.045	.427	.124	.285	.120	.385
Health	.531	.518	.624	.493	.527	.531	.528	.531	.539
Access to health services	.966	.911	.958	.970	.823	.966	.905	.967	.848
GP Prescriptions: Hypertension	.661	.668	.702	.644	.615	.662	.670	.662	.615
GP Prescriptions: Diabetes	.371	.379	.647	.257	.461	.372	.400	.370	.468
GP Prescriptions: Antidepressants	.432	.376	.570	.377	.411	.433	.395	.433	.396
GP Prescriptions: Obesity	.362	.292	.538	.291	.538	.362	.321	.362	.547
GP Prescriptions: Dementia	.490	.581	.398	.525	.421	.489	.570	.488	.459
Education and Learning	.866	.742	.879	.864	.641	.866	.743	.868	.627
Access to Schools	.937	.730	.930	.945	.446	.937	.718	.941	.408

Access to High Quality Schools	.999	.963	1.000	.999	.780	.999	.965	1.000	.757
Access to Adult Education	.895	.793	.857	.913	.777	.895	.791	.897	.798
Access to Libraries	.901	.787	.900	.905	.720	.901	.789	.903	.730
Culture, Heritage and Leisure	.711	.577	.726	.708	.589	.711	.593	.713	.572
Places of Worship	.932	.778	.908	.945	.678	.932	.781	.935	.664
Presence of Artists and Musicians	.349	.265	.472	.300	.565	.349	.305	.349	.533
Leisure Facilities	.930	.912	.906	.941	.820	.930	.909	.931	.827
Museums, Galleries, Music Halls and Theatres	.815	.696	.774	.834	.683	.815	.700	.817	.682
Listed Buildings	.537	.265	.574	.529	.234	.537	.298	.541	.192
Housing, Space and Environment	.512	.599	.579	.483	.547	.512	.601	.510	.547
Affordability of Housing	.861	.882	.779	.894	.796	.860	.863	.861	.813
Overcrowding	.314	.542	.618	.184	.608	.316	.571	.310	.585
Access to Green Space	.855	.719	.851	.861	.364	.855	.714	.858	.399
Access to Public Space	.877	.723	.873	.883	.648	.877	.717	.880	.654
Air Quality	.184	.297	.254	.153	.304	.184	.297	.182	.281
Pollution	.268	.614	.305	.244	.818	.267	.625	.260	.813
Transport, Mobility and Connectivity	.758	659	.754	.763	.558	.759	.662	.760	.569
Internet Provision	.691	.623	.707	.687	.451	.691	.630	.693	.463
Public Transport	.680	.569	.656	.692	.559	.680	.568	.682	.567

Adjusted Analysis (Main Analysis Adjusted for Gender and Ethnicity)

Table S3. Associations Between Aspects of Community Wellbeing and Mental Health Trajectories, Adjusting for Gender and Ethnicity

		Life Satisfaction Trajectory [a] OR [95% CI]			Emotional Difficulties Trajectory [b] OR [95% CI]	
Pathway	Predictor	Improving	Deteriorating	Low	Sub-clinical	Worsening
b^1	Relationships and Trust	.981 [.798, 1.207]	.916 [.740, 1.133]	1.068 [.930, 1.227]	1.092 [.968, 1.233]	1.032 [.891, 1.197]
b^2	Equality	.962 [.845, 1.093]	1.052 [.920, 1.203]	1.079 [.979, 1.189]	.972 [.905, 1.045]	.994 [.894, 1.104]
b^3	Voice and Participation	.942 [.840, 1.057]	1.024 [.867, 1.209]	1.000 [.916, 1.093]	1.073 [.998, 1.153]	.964 [.848, 1.095]
b^4	Economy, Work and Employment	.815 [.648, 1.024]	1.052 [.819, 1.354]	1.052 [.906, 1.223]	.966 [.850, 1.101]	1.130 [.929, 1.373]
b^5	Health	1.016 [.918, 1.123]	1.047 [.953, 1.151]	.979 [.915, 1.047]	.969 [.929, 1.012]	1.045 [.949, 1.149]
b^6	Education and Learning	.942 [.788, 1.126]	1.171 [.949, 1.445]	.891 [.785, 1.012]	.984 [.885, 1.094]	.894 [.768, 1.041]
b^7	Culture, Heritage and Leisure	.943 [.819, 1.089]	1.082 [.917, 1.279]	1.121* [1.015, 1.237]	.967 [.899, 1.042]	1.104 [.967, 1.261]
b^8	Housing, Space and Environment	.933 [.781, 1.114]	.883 [.723, 1.078]	1.093 [.972, 1.229]	1.100 [.986, 1.226]	1.050 [.907, 1.218]
b^9	Transport, Mobility and Connectivity	1.051 [.846, 1.307]	.775* [.602, .998]	.953 [.815, 1.114]	1.124 [.988, 1.280]	1.088 [.889, 1.332]

[a] Reference class: Consistently High life satisfaction, [b] Reference class: Few emotional difficulties, * Statistically significant

Table S4. Total, Direct and Indirect Effects of Neighbourhood Deprivation and Community Wellbeing on Life Satisfaction Trajectories, Adjusting for Gender and Ethnicity

		Life Satisfaction Trajectory [a]		
Pathway	Mediator	Improving	Deteriorating	Low
Total effect (before inclusion of mediators)				
c OR [95% CI]	*Class Regressed on Neighbourhood IMD*	.965 [.925, 1.006]	1.061 [1.006, 1.119] *	1.124 [1.085, 1.165] *
Direct effect (adjusted for mediators)				
c′[1] OR [95% CI]	*Class Regressed on Neighbourhood IMD*	.891 [.829, .960] *	1.082 [.981, 1.194]	1.147 [1.082, 1.215] *
*Indirect (mediated) effect (product of paths a*b)*				
α*b*[1] B [95% CI]	*Rel. & Trust*	.000 [−.005, .005]	−.002 [−.008, .004]	.002 [−.003, .006]
α*b*[2] B [95% CI]	*Equality*	−.005 [−.020, .011]	.006 [−.010, .022]	.009 [−.003, .022]
α*b*[3] B [95% CI]	*Voice & Part.*	.003 [−.003, .009]	−.001 [−.009, .007]	.000 [−.004, .004]
α*b*[4] B [95% CI]	*Ec., Work & Empl.*	.053 [−.007, .114]	−.013 [−.079, .052]	−.013 [−.053, .026]
α*b*[5] B [95% CI]	*Health*	−.003 [−.020, .015]	−.008 [−.024, .009]	.004 [−.008, .015]
α*b*[6] B [95% CI]	*Edu. & Learn.*	−.004 [−.017, .009]	.011 [−.005, .027]	−.008 [−.018, .002]
α*b*[7] B [95% CI]	*Cult., Heri. & Leis.*	−.001 [−.004, .003]	.001 [−.004, .006]	.001 [−.005, .008]
α*b*[8] B [95% CI]	*Hous, Spac. & Env.*	.012 [−.018, .042]	.021 [−.013, .055]	−.015 [−.035, .005]
α*b*[9] B [95% CI]	*Trans., Mob. & Conn.*	.005 [−.018, .029]	−.028 [−.060, .004]	−.005 [−.022, .012]
Covariates of Trajectory Class Membership				
OR [95% CI]	*Gender (Boys; Girls as reference)*	.594 [.486, .727] *	.807 [.645, 1.010]	.205 [.168, .250] *
OR [95% CI]	*Ethnicity (Asian; White as reference)*	1.218 [.659, 2.248]	.930 [.501, 1.727]	1.273 [.875, 1.852]
OR [95% CI]	*Ethnicity (Black; White as reference)*	1.197 [.938, 1.526]	1.238 [.936, 1.638]	.518 [.409, .655] *
OR [95% CI]	*Ethnicity (Chinese; White as reference)*	1.009 [.653, 1.558]	.886 [.558, 1.407]	.863 [.637, 1.171]
OR [95% CI]	*Ethnicity (Mixed; White as reference)*	1.561 [.601, 4.056]	1.380 [.618, 3.078]	.783 [.304, 2.021]
OR [95% CI]	*Ethnicity (AOEG; White as reference)*	1.446 [1.021, 2.049] *	1.095 [.733, 1.635]	1.004 [.779, 1.295]

[a] Reference class: Consistently High life satisfaction, [b] Reference class: Few emotional difficulties, * Statistically significant, *AOEG* Any Other Ethnic Group. Results adjusted for Gender and Ethnicity.

Table S5. Total, Direct and Indirect Effects of Neighbourhood Deprivation and Community Wellbeing on Emotional Difficulties Trajectories, Adjusting for Gender and Ethnicity

Pathway	Mediator	Emotional Difficulties Trajectory [b] Sub-clinical	Worsening
Total effect (before inclusion of mediators)			
c OR [95% CI]	*Class Regressed on Neighbourhood IMD*	1.013 [.990, 1.037]	1.027 [.980, 1.077]
Direct effect (adjusted for mediators)			
c'[1] OR [95% CI]	*Class Regressed on Neighbourhood IMD*	1.012 [.969, 1.058]	1.084 [1.006, 1.169] *
*Indirect (mediated) effect (product of paths a*b)*			
αb^1 B [95% CI]	*Rel. & Trust*	.002 [–.002, .006]	.001 [–.003, .004]
αb^2 B [95% CI]	*Equality*	–.003 [–.012, .005]	–.001 [–.013, .012]
αb^3 B [95% CI]	*Voice & Part.*	–.003 [–.008, .001]	.002 [–.005, .008]
αb^4 B [95% CI]	*Ec., Work & Empl.*	.009 [–.025, .043]	–.032 [–.084, .020]
αb^5 B [95% CI]	*Health*	.005 [–.002, .013]	–.008 [–.024, .009]
αb^6 B [95% CI]	*Edu. & Learn.*	–.001 [–.009, .006]	–.008 [–.020, .004]
αb^7 B [95% CI]	*Cult., Heri. & Leis.*	.000 [–.003, .002]	.001 [–.004, .007]
αb^8 B [95% CI]	*Hous, Spac. & Env.*	–.016 [–.035, .003]	–.008 [–.034, .017]
αb^9 B [95% CI]	*Trans., Mob. & Conn.*	.013 [–.003, .028]	.009 [–.013, .031]
Covariates of Trajectory Class Membership			
OR [95% CI]	*Gender (Boys; Girls as reference)*	.130 [.116, .146] *	.183 [.150, .224] *
OR [95% CI]	*Ethnicity (Asian; White as reference)*	.535 [.394, .727] *	.840 [.544, 1.296]
OR [95% CI]	*Ethnicity (Black; White as reference)*	.427 [.360, .505] *	.428 [.332, .550] *
OR [95% CI]	*Ethnicity (Chinese; White as reference)*	.471 [.371, .598] *	.460 [.331, .639] *
OR [95% CI]	*Ethnicity (Mixed; White as reference)*	1.216 [.638, 2.320]	.795 [.337, 1.875]
OR [95% CI]	*Ethnicity (AOEG; White as reference)*	.627 [.507, .775] *	.733 [.540, .995] *

[a] Reference class: Consistently High life satisfaction, [b] Reference class: Few emotional difficulties, * Statistically significant, *AOEG* Any Other Ethnic Group. Results adjusted for Gender and Ethnicity.

Model Fit Criteria

Table S6. Model Fit Statistics for Enumeration of Life Satisfaction Trajectories (N = 27,009)

Model	Classes	Log(L)	AIC	BIC	ABIC	LMRa (*P*)	Entropy	Class Proportions Based on Estimated Posterior Probabilities (%)
**Life Satisfaction (Equal Within Group Variance)**								
Linear	1	-113,033	226,083	226,148	226,123	-	-	100
	2	-111,954	223,931	224,021	223,986	.000	.637	78, 22
	3	-111,449	222,926	223,041	222,996	.000	.655	73, 19, 8
	4	-111,141	222,317	222,457	222,403	.000	.659	71, 13, 9, 7
	5	-110,858	221,756	221,920	221,856	.001	.633	54, 29, 7, 6, 4
	6	-110,701	221,448	221,637	221,564	.000	.631	56, 22, 9, 5, 5, 3
	7	-110,498	221,048	221,262	221,179	.008	.635	33, 28, 18, 10, 4, 4, 3
	8	-110,315	220,689	220,927	220,835	.229	.679	32, 27, 18, 10, 4, 4, 3, 2
	9	-110,131	220,327	220,590	220,488	.000	.668	31, 27, 18, 7, 5, 4, 3, 3, 2
	10	-109,972	220,015	220,302	220,190	.000	.673	30, 27, 17, 6, 5, 5, 3, 3, 2, 2
Non-Linear	1	-113,020	226,059	226,133	226,104	-	-	100
	2	-111,931	223,887	223,986	223,947	.000	.621	78, 22
	3	-111,429	222,889	223,012	222,964	.010	.653	72, 20, 8
	4	-111,068	222,173	222,320	222,263	.002	.661	70, 15, 8, 7
	5	-110,730	221,502	221,674	221,608	.000	.635	33, 30, 21, 12, 4
	6	-110,407	220,863	221,060	220,983	.000	.674	33, 30, 19, 8, 6, 4
	7	-110,286	220,626	220,848	220,762	.004	.648	33, 28, 18, 11, 4, 4, 2
	8	-110,037	220,135	220,382	220,286	.010	.680	32, 28, 17, 9, 6, 3, 3, 2
	9	-109,862	219,791	220,061	219,956	.000	.675	31, 28, 17, 6, 5, 5, 3, 3, 2
	10	-109,687	219,446	219,741	219,627	.000	.678	30, 27, 17, 6, 5, 5, 4, 2, 2, 2
**Life Satisfaction (Zero Within Group Variance)**								
Linear	1	-113,094	226,200	226,250	226,230	-	-	100
	2	-112,128	224,275	224,349	224,320	.000	.660	79,21
	3	-111,458	222,941	223,039	223,001	.000	.653	73, 19, 9
	4	-111,146	222,323	222,446	222,398	.000	.661	71, 14, 8, 7
	5	-110,916	221,869	222,017	221,960	.000	.634	55, 28, 7, 7, 4
	6	-110,764	221,570	221,743	221,676	.005	.626	56, 19, 12, 5, 5, 3
	7	-110,668	221,385	221,582	221,506	.000	.617	52, 19, 13, 6, 6, 3, 3
	8	-110,471	220,996	221,218	221,132	.004	.641	32, 28, 17, 9, 6, 6, 4, 2
	9	-110,305	220,671	220,917	220,822	.002	.645	31, 27, 18, 7, 5, 5, 3, 2, 2
	10	-110,082	220,231	220,502	220,397	.000	.669	30, 26, 17, 6, 6, 5, 5, 4, 3, 2
Non-Linear	1	-113,082	226,179	226,237	226,215	-	-	100
	2	-112,124	224,268	224,350	224,318	.017	.653	79, 21
	3	-111,435	222,897	223,003	222,962	.000	.652	72, 20, 8
	4	-111,126	222,285	222,416	222,365	.000	.659	71, 14, 9, 6
	5	-110,788	221,615	221,771	221,711	.000	.642	55, 28, 7, 7, 3
	6	-110,609	221,262	221,442	221,372	.000	.644	57, 23, 9, 6, 5, 2
	7	-110,391	220,832	221,037	220,957	.001	.636	33, 28, 17, 10, 5, 4, 3
	8	-110,165	220,386	220,616	220,527	.000	.649	32, 28, 17, 10, 5, 3, 3, 2
	9	-109,955	219,972	220,227	220,128	.000	.651	29, 27, 13, 10, 7, 5, 4, 4, 2
	10	-109,722	219,512	219,791	219,683	.000	.673	30, 27, 17, 6, 6, 5, 5, 4, 2, 2

Table S7. Model Fit Statistics for Enumeration of Emotional Difficulties Trajectories (N = 26,461)

Model	Classes	Log(L)	AIC	BIC	ssaBIC	LMRa (*p*)	Entropy	Proportion of sample in smallest class (%)
			**Emotional Difficulties (Equal Within Group Variance)**					
Linear	1	-139,851	279,718	279,784	279,758	-	-	100
	2	-139,038	278,099	278,189	278,154	.000	.585	70, 30
	3	-138,378	276,785	276,899	276,855	.000	.615	53, 38, 8
	4	-137,998	276,030	276,170	276,115	.043	.642	49, 36, 11, 4
	5	-137,664	275,368	275,532	275,468	.000	.599	37, 27, 25, 9, 3
	6	-137,535	275,116	275,305	275,232	.000	.628	36, 26, 24, 9, 2, 2
	7	-137,364	274,780	274,993	274,910	.024	.587	36, 26, 13, 13, 13, 8, 2
	8	-137,184	274,426	274,663	274,571	.002	.606	35, 25, 13, 12, 8, 2, 2, 2
	9	-137,031	274,127	274,389	274,287	.001	.592	29, 20, 18, 10, 10, 7, 2, 2, 2
	10	-136,920	273,910	274,197	274,086	.158	.595	29, 20, 14, 11, 9, 5, 4, 4, 2, 2
Non-Linear	1	-139,841	279,700	279,774	279,745	-	-	100
	2	-139,026	278,076	278,175	278,136	.000	.584	70, 30
	3	-138,287	276,605	276,728	276,680	.000	.612	54, 38, 8
	4	-137,820	275,677	275,824	275,767	.000	.666	51, 38, 9, 2
	5	-137,465	274,972	275,144	275,077	.000	.595	37, 27, 25, 9, 2
	6	-137,275	274,598	274,795	274,719	.000	.626	36, 27, 24, 9, 3, 1
	7	-137,142	274,338	274,559	274,473	.000	.632	45, 27, 9, 6, 6, 5, 2
	8	-136,912	273,885	274,130	274,035	.000	.608	35, 25, 14, 11, 8, 3, 2, 2
	9	-136,798	273,663	273,933	273,828	.094	.620	34, 34, 8, 7, 7, 3, 2, 2, 3
	10	-136,654	273,381	273,676	273,562	.211	.614	32, 18, 6, 5, 5, 5, 4, 4, 4, 17
			**Emotional Difficulties (Zero Within Group Variance)**					
Linear	1	-139,937	279,886	279,935	279,916	-	-	100
	2	-139,148	278,314	278,388	278,359	.000	.598	71, 29
	3	-138,580	277,184	277,283	277,244	.000	.656	68, 27, 5
	4	-138,225	276,481	276,604	276,556	.000	.664	51, 35, 10, 4
	5	-137,922	275,881	276,028	275,971	.001	.642	47, 33, 10, 8, 2
	6	-137,658	275,359	275,531	275,464	.247	.620	46, 27, 11, 7, 5, 4
	7	-137,430	274,909	275,105	275,029	.126	.591	36, 23, 15, 13, 6, 3, 4
	8	-137,248	274,550	274,771	274,685	.001	.601	37, 23, 13, 12, 6, 5, 2, 2
	9	-137,143	274,347	274,593	274,498	.086	.604	36, 22, 13, 11, 7, 5, 2, 2, 2
	10	-137,042	274,150	274,420	274,316	.056	.598	35, 18, 12, 11, 9, 5, 3, 3, 2, 2
Non-Linear	1	-139,928	279,870	279,927	279,905	-	-	100
	2	-139,100	278,220	278,301	278,270	.000	.589	71, 29
	3	-138,466	276,959	277,065	277,024	.000	.681	68, 28, 4
	4	-138,082	276,196	276,327	276,276	.000	.684	51, 36, 10, 3
	5	-137,658	275,354	275,510	275,449	.000	.659	47, 34, 9, 6, 3
	6	-137,347	274,739	274,919	274,849	.000	.618	48, 24, 15, 7, 4, 2
	7	-137,198	274,447	274,652	274,572	.017	.590	38, 24, 16, 10, 6, 4, 2
	8	-137,003	274,062	274,291	274,202	.002	.605	34, 24, 15, 11, 8, 4, 3, 1
	9	-136,835	273,733	273,986	273,888	.002	.619	33, 24, 17, 8, 7, 4, 4, 2, 1
	10	-136,669	273,407	273,686	273,578	.002	.593	29, 19, 12, 12, 12, 4, 4, 4, 2, 2

***a* and *b* Pathways for Main Analysis**

Table S8. Standardised Regression Coefficients Between Neighbourhood Deprivation and Aspects of Community Wellbeing with 95% Confidence Intervals

Pathway	Community Wellbeing Regressed on Neighbourhood Deprivation:	Life Satisfaction Model	Emotional Difficulties Model
α^1	Relationships and Trust	.078 [−.040, .196]	.078 [-.041, .196]
α^2	Equality	.268 * [.154, .382]	.268 * [.155, .382]
α^3	Voice and Participation	−.136 * [−.268, −.003]	-.136 * [-.268, -.003]
α^4	Economy, Work and Employment	−.739 * [−.795, −.684]	-.740 * [-.795, -.685]
α^5	Health	−.329 * [−.430, −.227]	-.329 * [-.430, -.227]
α^6	Education and Learning	.183 * [.062, .305]	.184 * [.063, .305]
α^7	Culture Heritage and Leisure	.031 [−.093, .155]	.031 [-.093, .155]
α^8	Housing, Space and Environment	−.453 * [−.549, −.358]	-.454 * [-.549, -.358]
α^9	Transport, Mobility and Connectivity	.245 * [.132, .359]	.245 * [.132, .359]

* Statistically significant.

Note: Coefficients are (almost) identical across the life satisfaction and emotional difficulties models, as both were estimated on the same underlying sample; excluding only participants with missing outcome data specific to each model. That is, missingness on emotional difficulties was not considered in the life satisfaction model, and vice versa. Results from both models are presented for completeness.

Table S9. Associations Between Aspects of Community Wellbeing and Mental Health Trajectories

		Life Satisfaction Trajectory [a] OR [95% CI]			Emotional Difficulties Trajectory [b] OR [95% CI]	
Pathway	Predictor	Improving	Deteriorating	Low	Sub-clinical	Worsening
b^1	Relationships and Trust	1.021 [.830, 1.258]	.945 [.768, 1.162]	.983 [.866, 1.117]	.993 [.893, 1.104]	.937 [.812, 1.081]
b^2	Equality	.940 [.811, 1.090]	1.035 [.896, 1.196]	1.139 * [1.035, 1.253]	1.046 [.978, 1.119]	1.056 [.947, 1.177]
b^3	Voice and Participation	.941 [.830, 1.066]	1.004 [.851, 1.184]	1.052 [.964, 1.148]	1.093 * [1.025, 1.166]	.989 [.870, 1.124]
b^4	Economy, Work and Employment	.824 [.643, 1.056]	1.056 [.816, 1.366]	1.054 [.906, 1.227]	.972 [.859, 1.100]	1.133 [.938, 1.368]
b^5	Health	1.001 [.897, 1.117]	1.039 [.942, 1.145]	.991 [.929, 1.057]	.967 [.927, 1.009]	1.043 [.950, 1.145]
b^6	Education and Learning	.937 [.779, 1.126]	1.162 [.943, 1.433]	.909 [.803, 1.029]	.974 [.887, 1.070]	.892 [.774, 1.027]
b^7	Culture, Heritage and Leisure	.937 [.801, 1.096]	1.071 [.903, 1.271]	1.145 * [1.033, 1.269]	.985 [.918, 1.057]	1.120 [.985, 1.273]
b^8	Housing, Space and Environment	.895 [.738, 1.085]	.858 [.700, 1.052]	1.163 * [1.043, 1.296]	1.169 * [1.060, 1.289]	1.105 [.959, 1.272]
b^9	Transport, Mobility and Connectivity	1.043 [.837, 1.300]	.771 * [.610, .976]	.943 [.805, 1.105]	1.101 [.976, 1.241]	1.067 [.868, 1.312]

[a] Reference class: Consistently High life satisfaction, [b] Reference class: Low/Lessening emotional difficulties, * Statistically significant

Mapping the Co-op Wellbeing Index Across Greater Manchester Seamless Locales

Figure S1. Heat map of Greater Manchester: Relationships and Trust

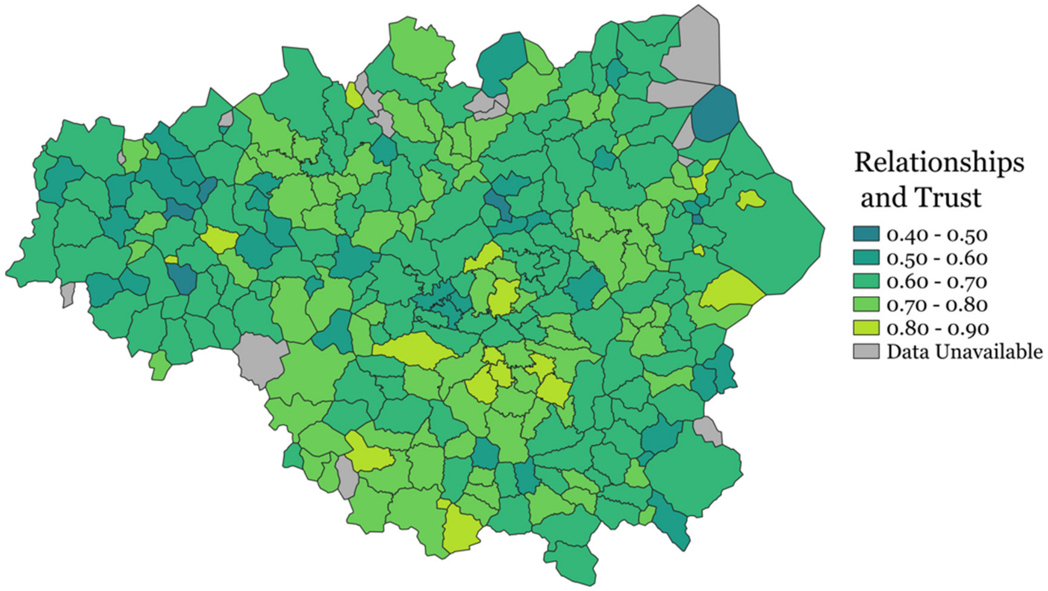

Figure S2. Heat map of Greater Manchester: Equality

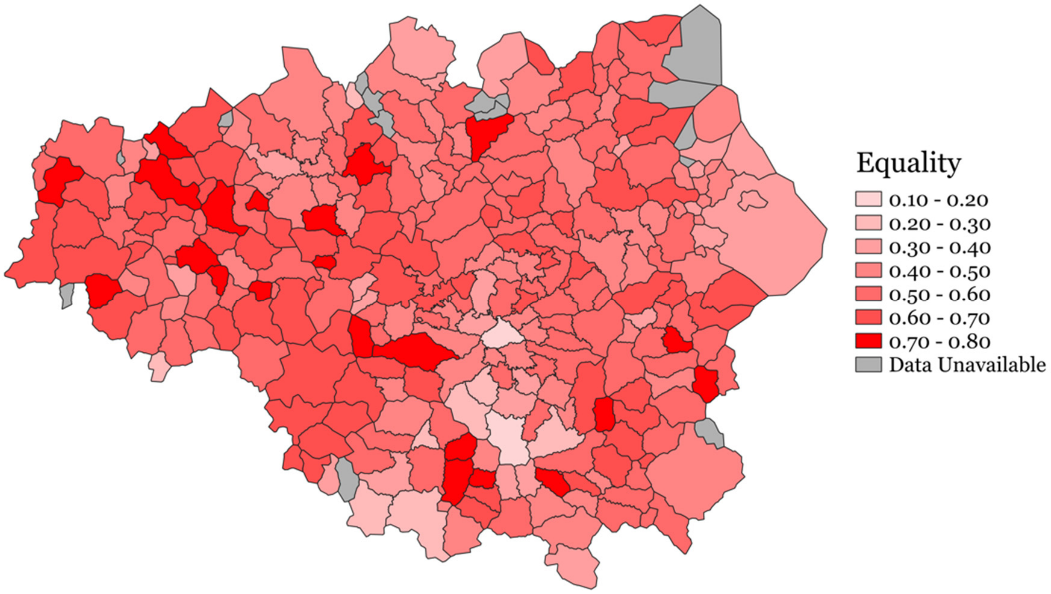

Figure S3. Heat map of Greater Manchester: Voice and Participation

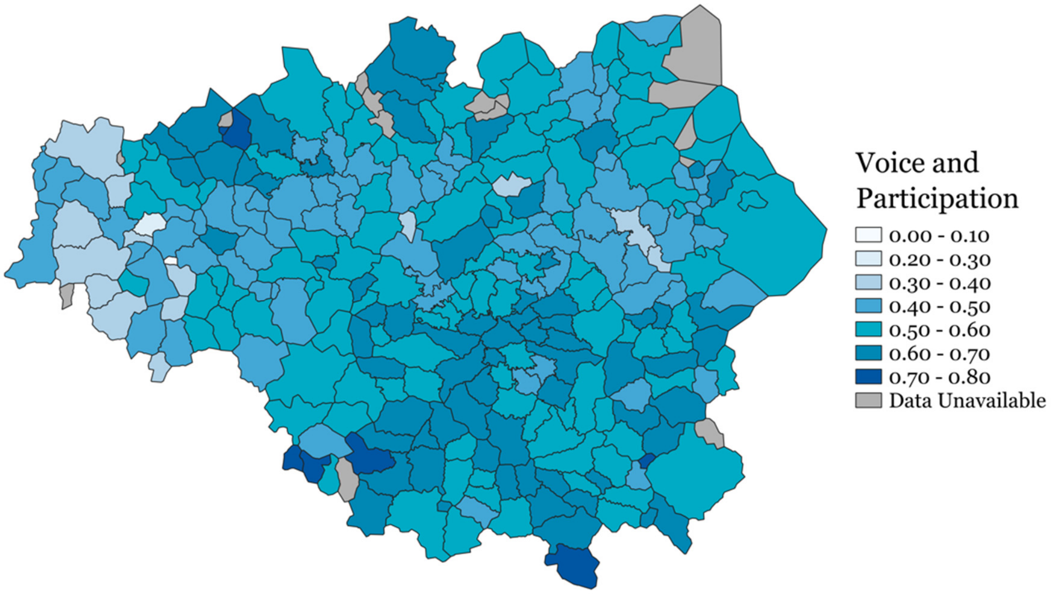

Figure S4. Heat map of Greater Manchester: Economy, Work and Employment

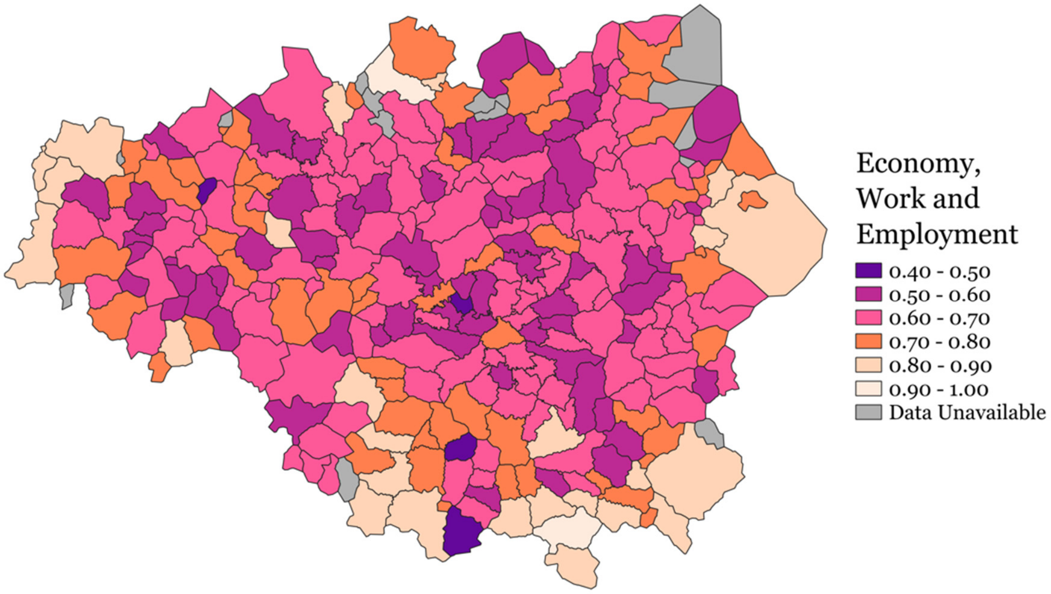

Figure S5. Heat map of Greater Manchester: Health

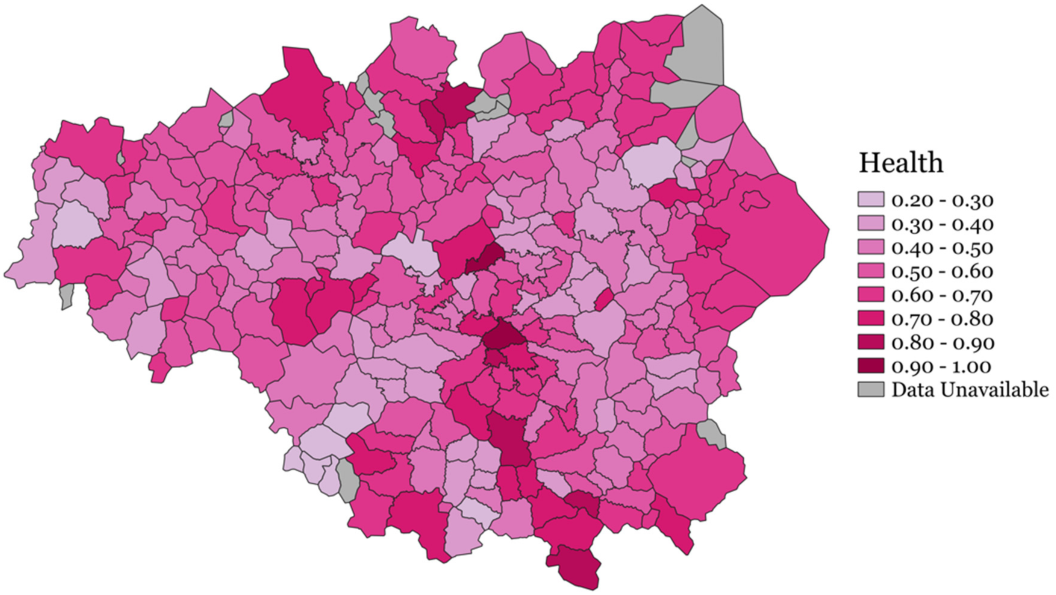

Figure S6. Heat map of Greater Manchester: Education and Learning

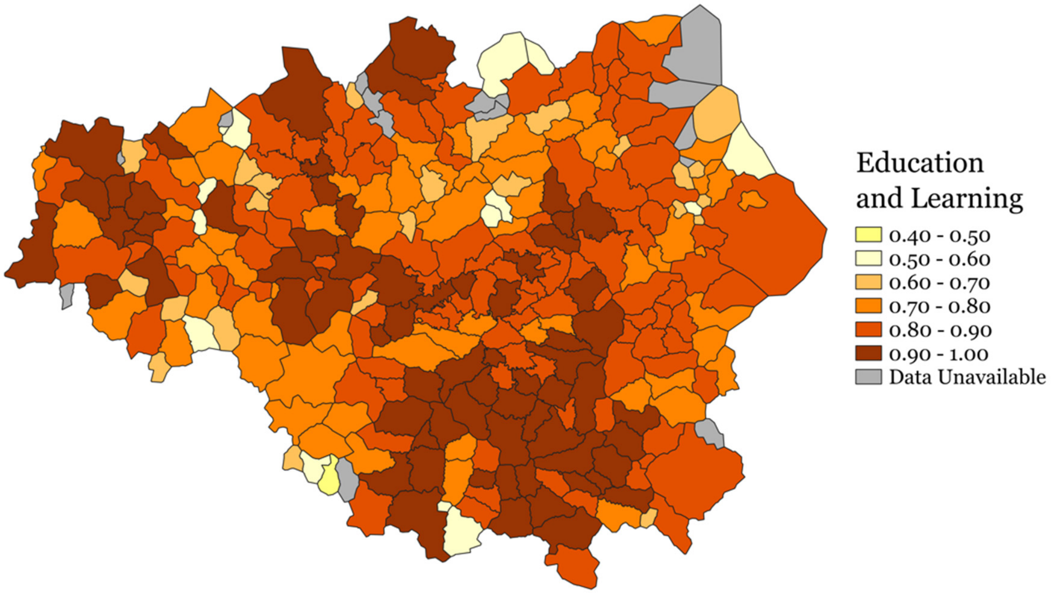

Figure S7. Heat map of Greater Manchester: Culture, Heritage and Leisure

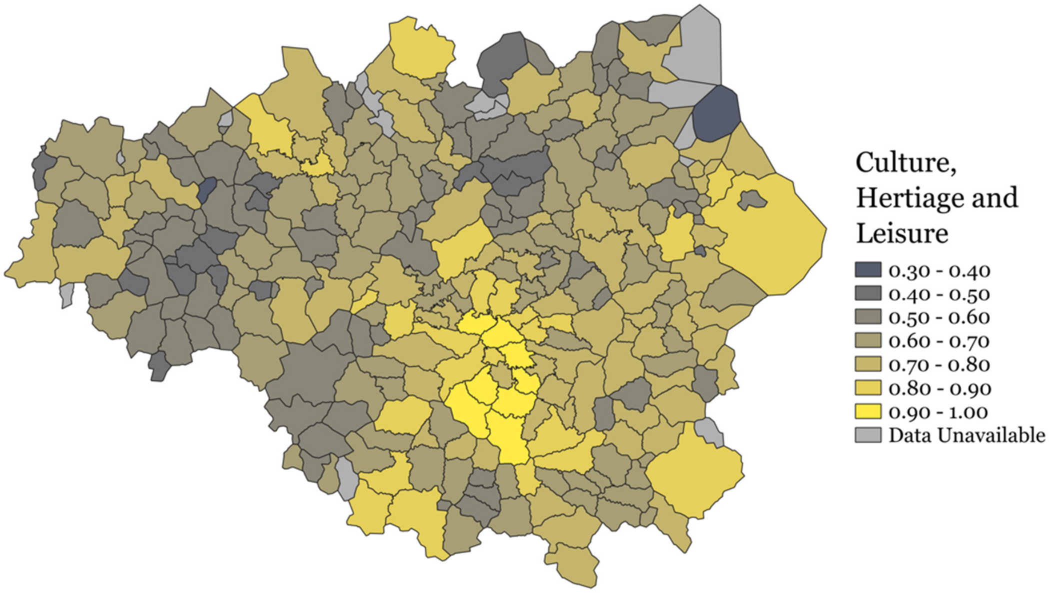

Figure S8. Heat map of Greater Manchester: Housing, Space and Environment

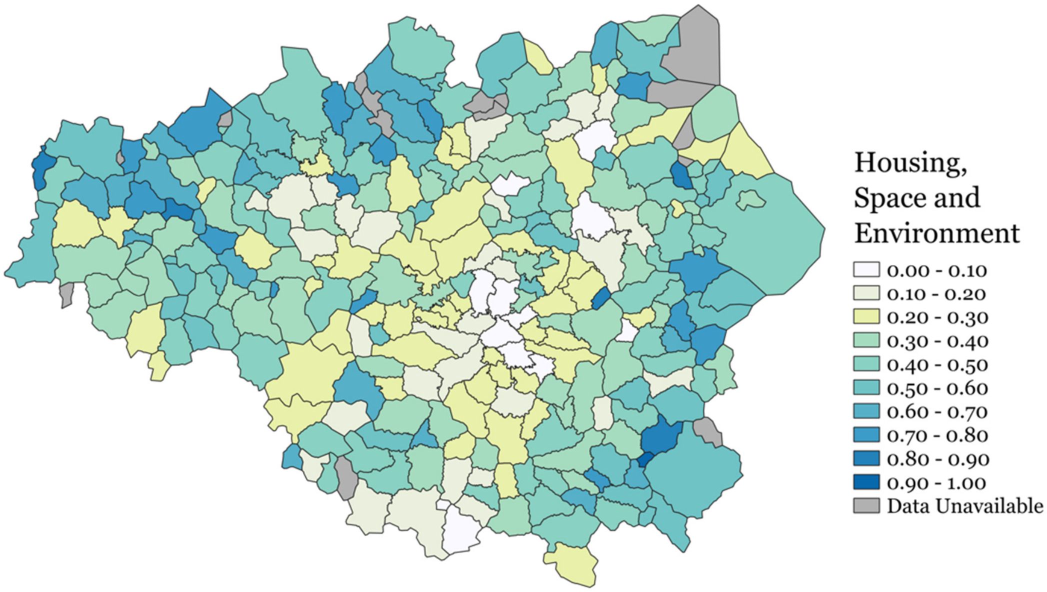

Figure S9. Heat map of Greater Manchester: Transport, Mobility and Connectivity

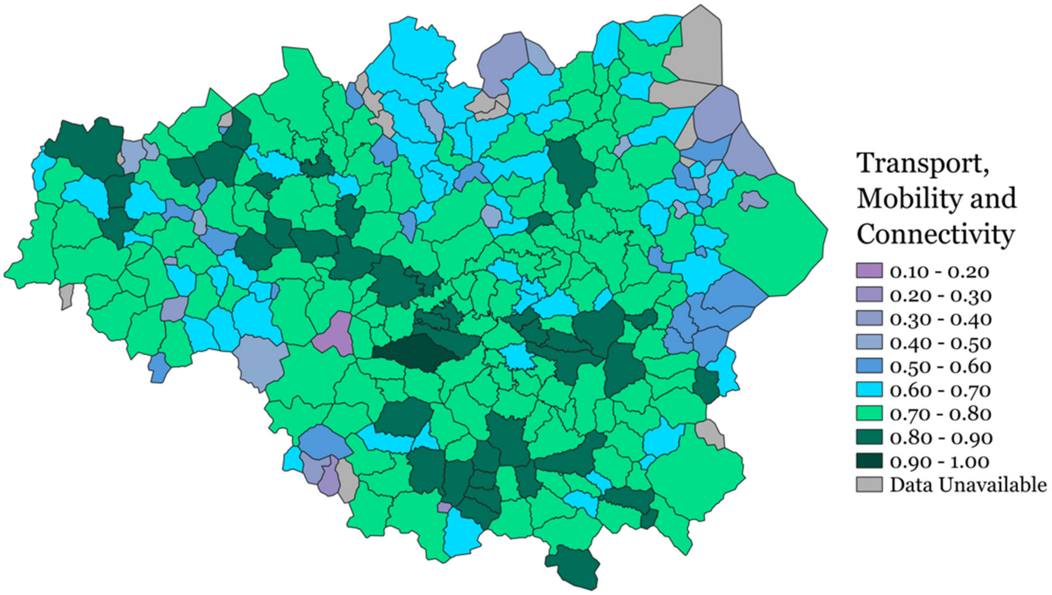

Alternative Model Solutions (Probability Plots)

Figure S10. Life Satisfaction - Linear Growth with Variance Held Equal Across Classes

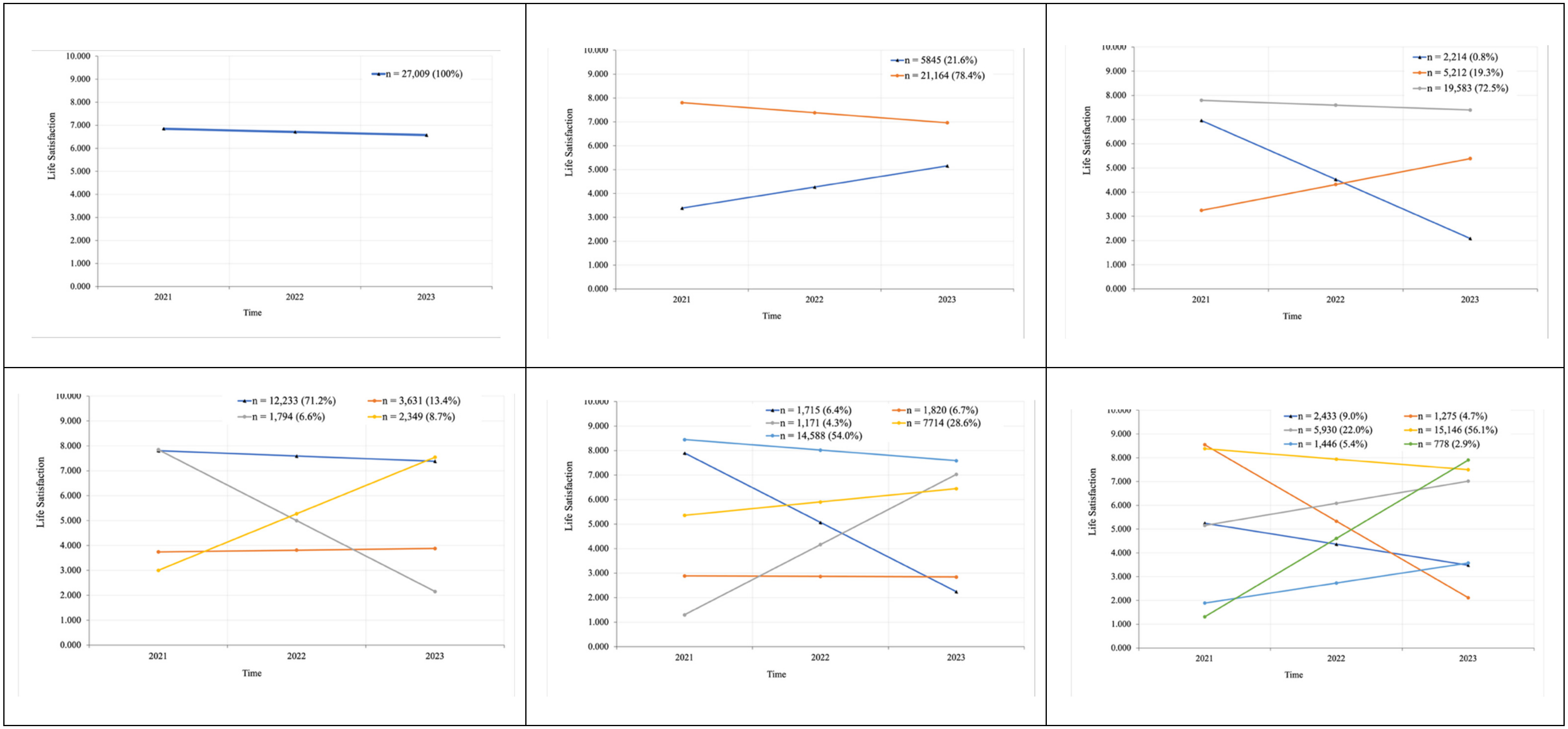

Figure S11. Life Satisfaction – Non-Linear Growth with Variance Held Equal Across Classes

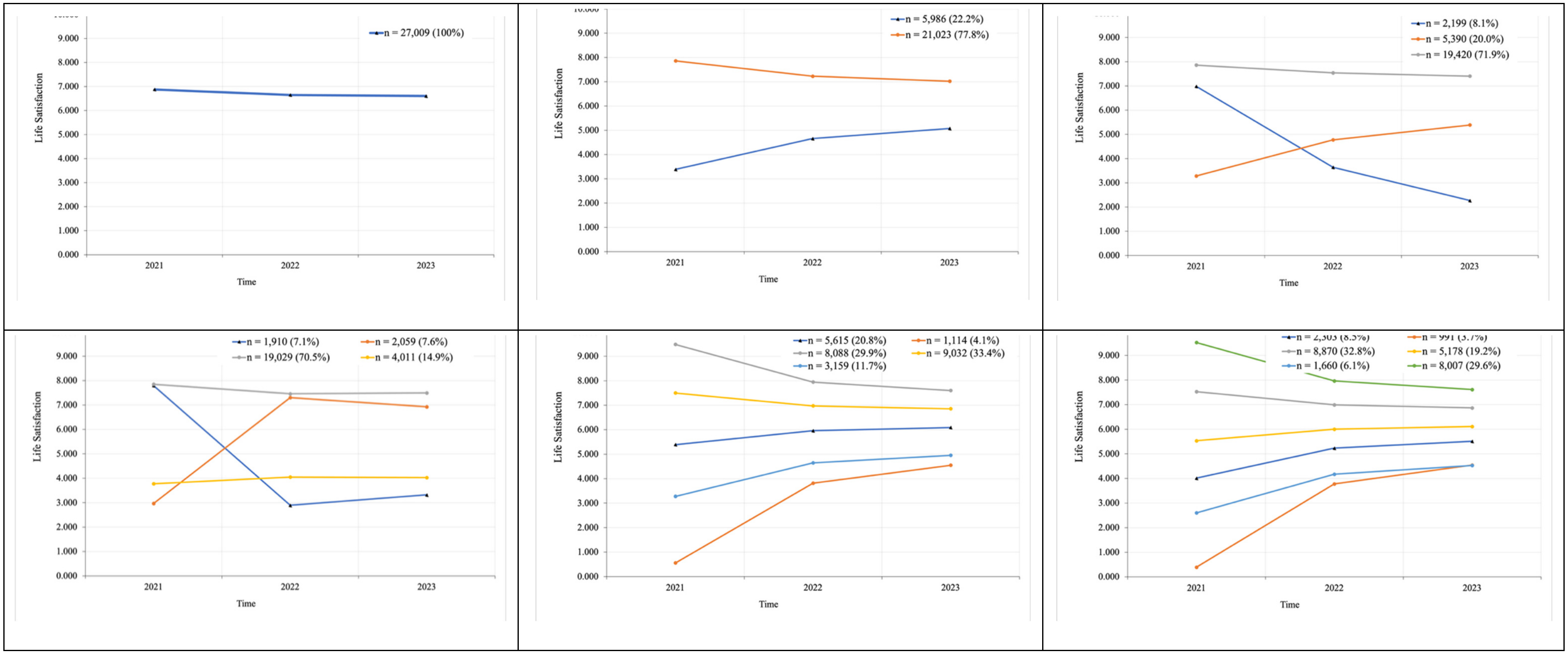

Figure S12. Life Satisfaction – Linear Growth with Variance of Slope Factor Fixed at Zero

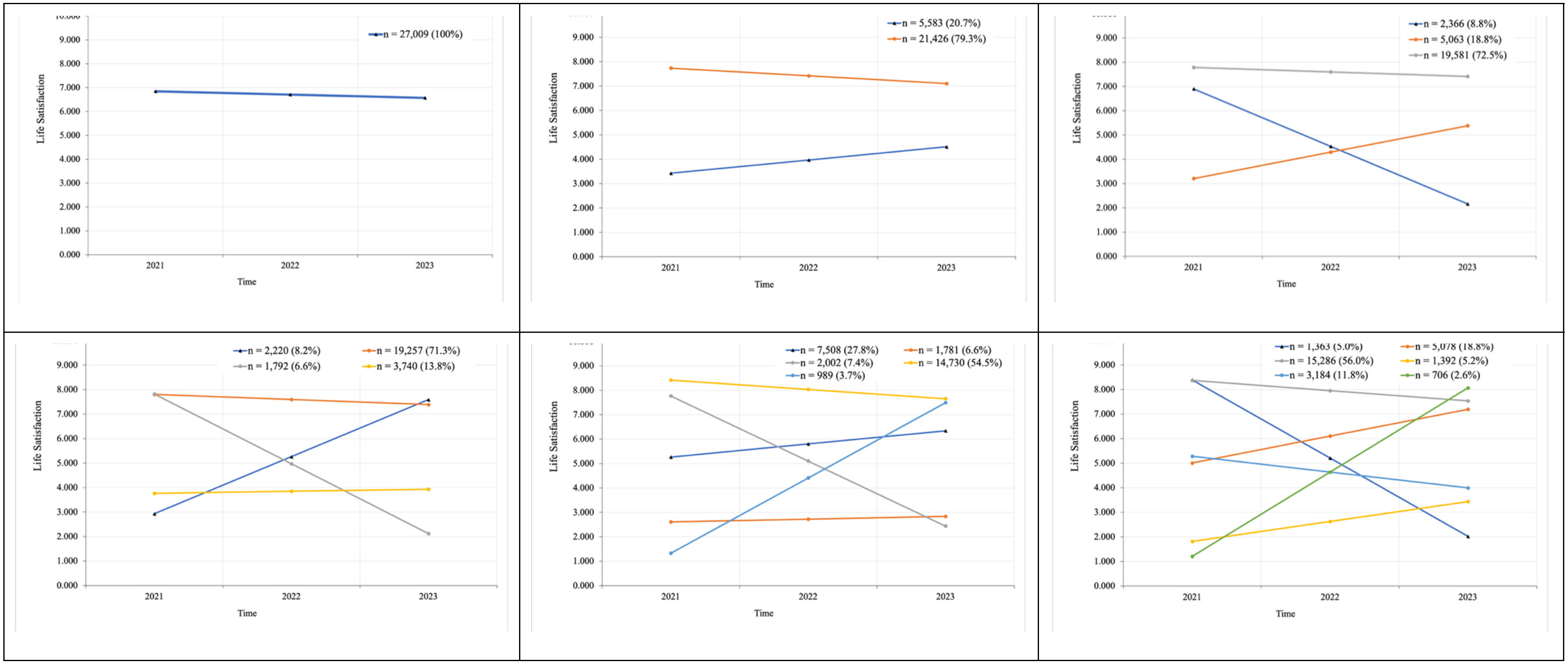

Figure S13. Life Satisfaction – Non-Linear Growth with Variance of Slope Factor Fixed at Zero

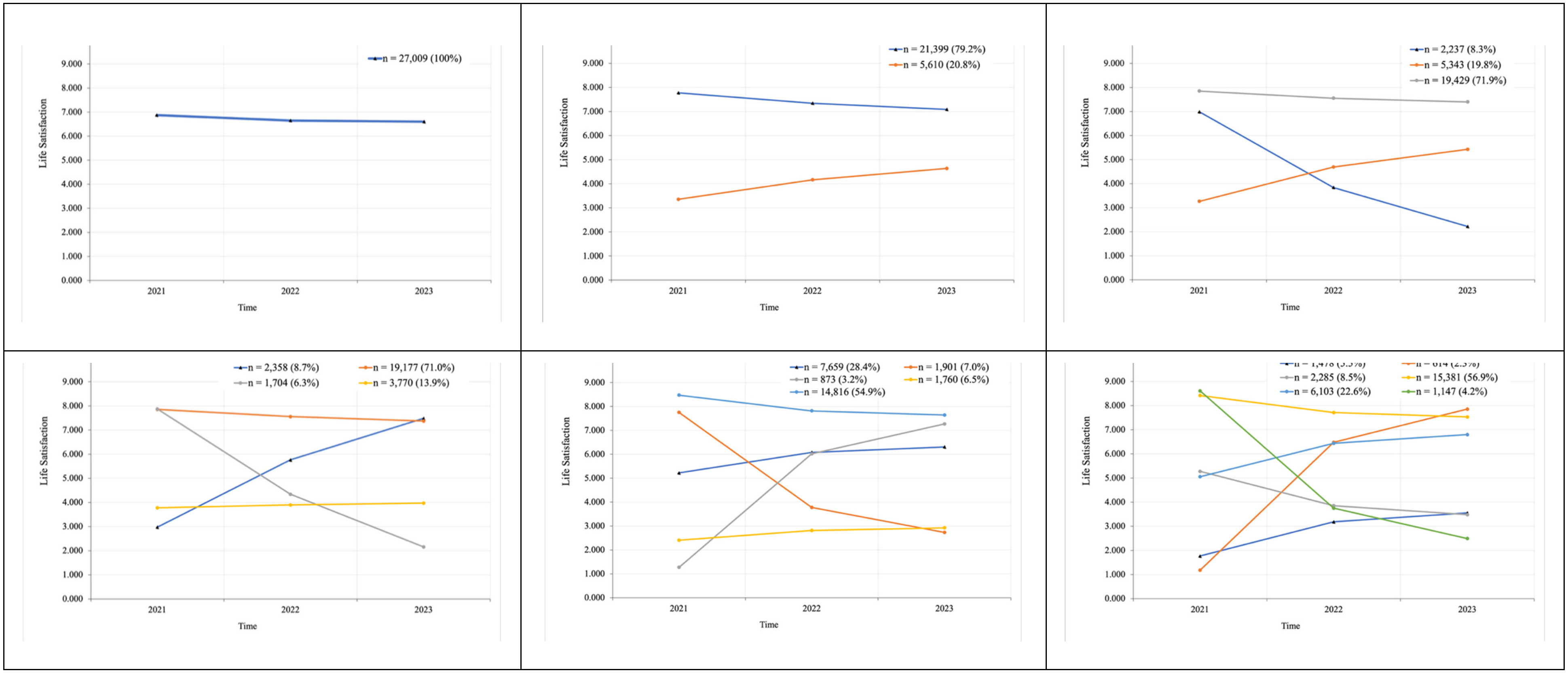

Figure S14. Emotional Difficulties - Linear Growth with Variance Held Equal Across Classes

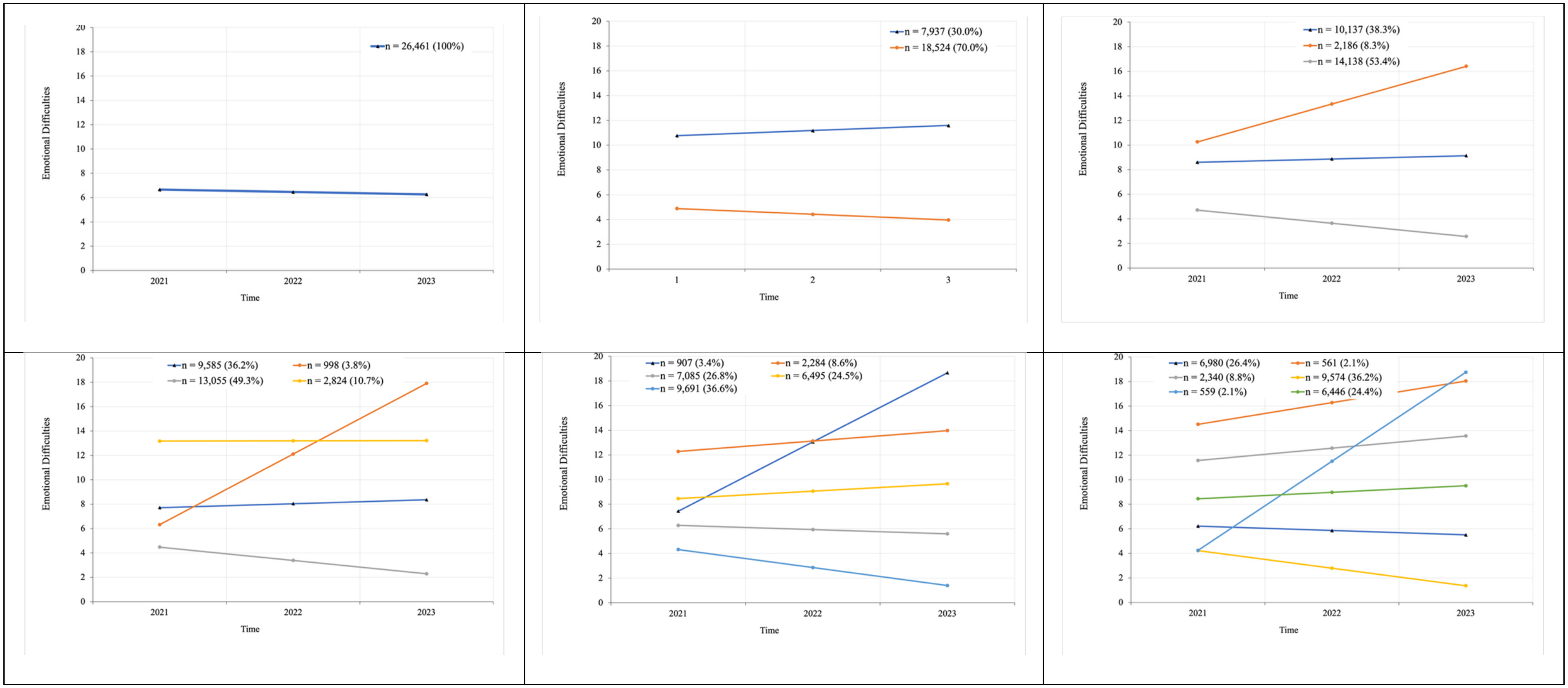

Figure S15. Emotional Difficulties – Non-Linear Growth with Variance Held Equal Across Classes

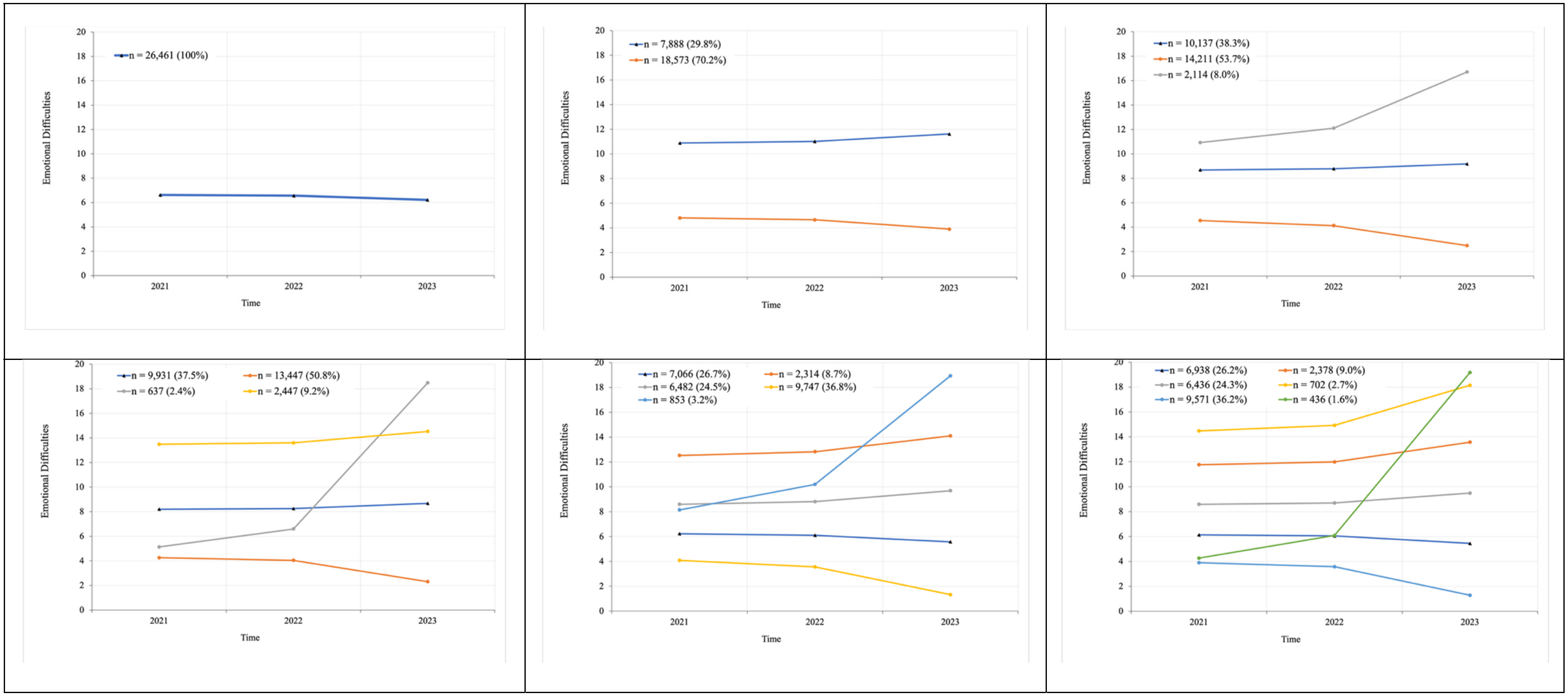

Figure S16. Emotional Difficulties – Linear Growth with Variance of Slope Factor Fixed at Zero

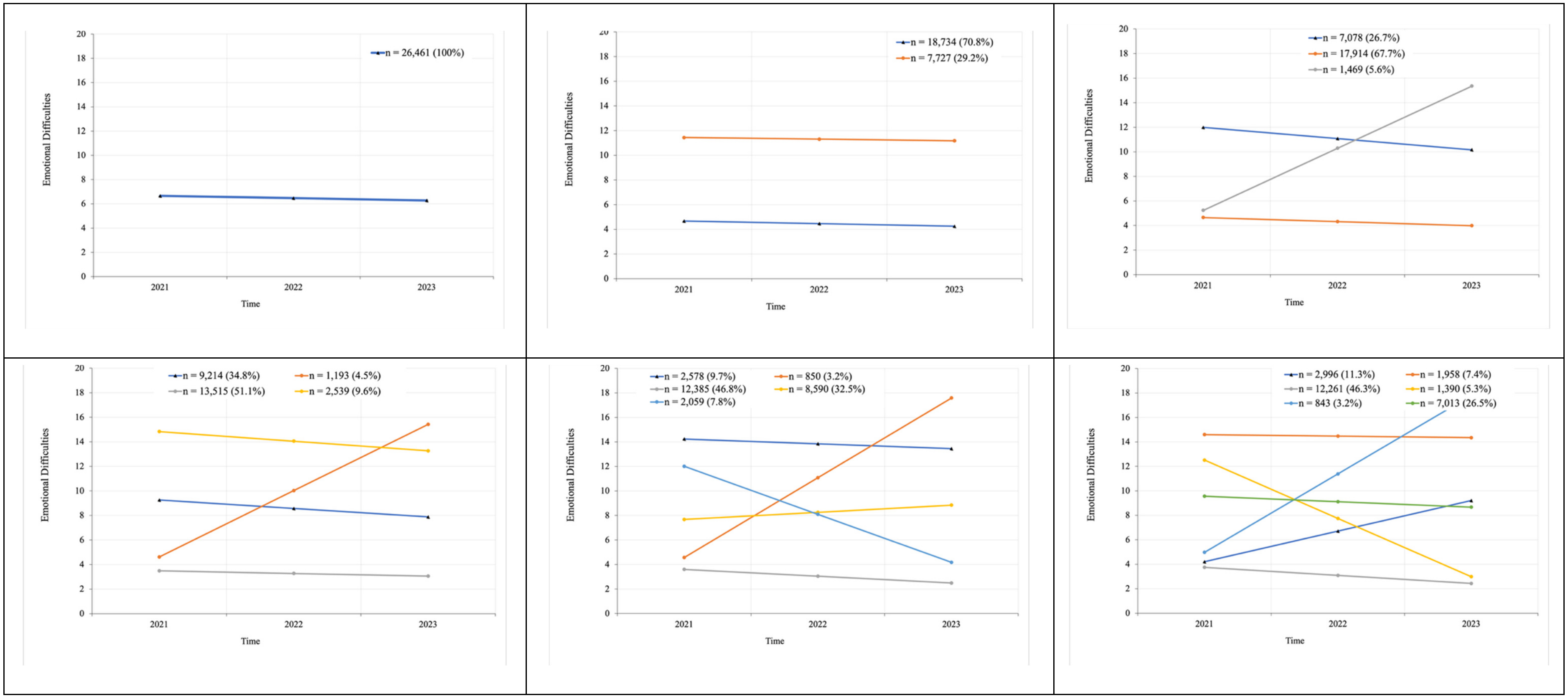

Figure S17. Emotional Difficulties – Non-Linear Growth with Variance of Slope Factor Fixed at Zero

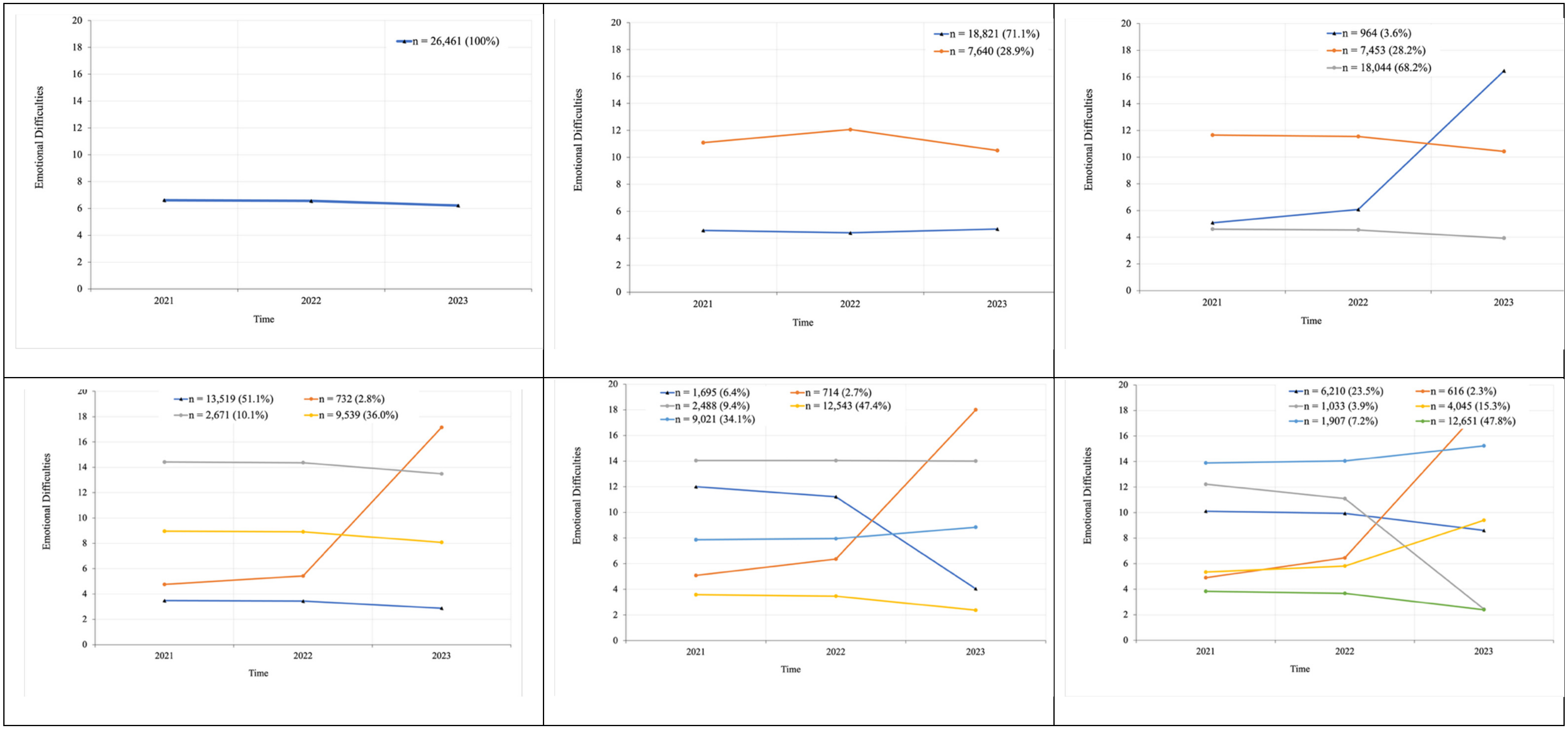

Supplement: Supplementary file 1 [file ijerph-22-00951-s001.zip › ijerph-3631430-supplementary.pdf]
